# Exercise Attenuates Brain Aging by Rescuing Down-Regulated Wnt/β-Catenin Signaling in Aged Rats

**DOI:** 10.3389/fnagi.2020.00105

**Published:** 2020-04-23

**Authors:** Dandan Chen, Ying Zhang, Meng Zhang, Jingru Chang, Zhenzhong Zeng, Xianjuan Kou, Ning Chen

**Affiliations:** Hubei Key Laboratory of Exercise Training and Monitoring, College of Health Science, Wuhan Sports University, Wuhan, China

**Keywords:** exercise, brain aging, Wnt, DKK-1, β-catenin

## Abstract

Down-regulated Wnt signaling is involved in brain aging with declined cognitive capacity due to its modulation on neuronal function and synaptic plasticity. However, the molecular mechanisms are still unclear. In the present study, the naturally aged rat model was established by feeding rats from 6 months old to 21 months old. The cognitive capacity of aged rats was compared with young rats as the controls and the aged rats upon 12-week exercise interventions including treadmill running, resistance exercise, and alternating exercise with resistance exercise and treadmill running. Wnt signaling was examined in hippocampal tissues of the rats from different groups. Results indicated that the expression of Dickkopf-1 (DKK-1) as an antagonist of Wnt signal pathway, the activation of GSK-3β, and the hyperphosphorylated Tau were markedly increased as the extension of age. Meanwhile, higher p-β-catenin^Ser33, 37, Thr41^ promoted neuronal degradation of aged rats. In contrast, three kinds of exercise interventions rescued the abnormal expression of DKK-1 and synaptophysin such as PSD-93 and PSD-95 in hippocampal tissues of the aged rats; especially 12-week treadmill running suppressed DKK-1 up-regulation, GSK-3β activation, β-catenin phosphorylation, and hyperphosphorylated Tau. In addition, the down-regulated PI3K/AKT and Wnt signal pathways were observed in aged rats, but could be reversed by resistance exercise and treadmill running. Moreover, the increased Bax and reduced Bcl-2 levels in hippocampal tissues of aged rats were also reversed upon treadmill running intervention. Taken together, down-regulated Wnt signaling suppressed PI3K/Akt signal pathway, aggravated synaptotoxicity, induced neuron apoptosis, and accelerated cognitive impairment of aged rats. However, exercise interventions, especially treadmill running, can attenuate their brain aging process *via* restoring Wnt signaling and corresponding targets.

## Introduction

Aging is the major risk factor for Alzheimer’s disease (AD). According to the latest report from United Nations, the population over 60 years old in the world is close to 1 billion in 2017. As the increase of life expectancy, population aging will be a serious issue for each country. Meanwhile, neurodegenerative diseases caused by aging are seriously threatening the quality of life of senior people. The number of suffered individuals could rise to more than 100 million by 2050 (Bayod et al., 2015). Current treatments cannot terminate or slow down the progression of AD due to unclear molecular mechanisms involved in AD pathogenesis. Therefore, developing an effective strategy to delay aging process and prevent the diseases associated with aging will be an urgent problem.

At present, different animal models such as natural aging animal models, oxidative stress-induced animal models, transgenic mice and senescence-accelerated mouse prone 8 (SAMP8) mice are used for exploring the pathogenesis and treatments of AD. The transgenic aging animal model mainly simulates the pathological aging by overexpressing APP and inducing PS1 gene mutation (Woodruff-Pak, 2008). Although the transgenic aging model can cause cognitive impairment in animals, the model usually lacks pathological changes of Tau protein in the body, but the model construction is complicated and expensive. The stress-induced aging model is prone to dose-dependent problems and large individual difference, and has certain requirements for experimental techniques. Compared with above two animal models, the spontaneous aging model has the problems with difficult acquisition due to long-term feeding and expensive feeding expenditure. However, the physiological change of the spontaneous aging model is close to aging process of humans. Therefore, in the present study, we used the spontaneous aging rat model for exploring accurate mechanisms of exercise for delaying aging process after overcoming a series of difficulties in long-term model construction. Wnt signaling is involved in learning and memory processes by regulating synaptic function and neuronal plasticity (Dickins and Salinas, 2013; Rosso and Inestrosa, 2013). Interestingly, recent evidence indicates that the impairment or defects in Wnt signaling is closely correlated with the pathogenesis of AD (Inestrosa et al., 2012). Dickkopf-1 (DKK-1) is an endogenous inhibitor to negatively regulate the canonical Wnt/β-catenin signal pathway. Similarly, the induction of DKK-1 accompanied by decreased number of synapses and neuronal viability is found in Aβ-induced neurons (Caricasole et al., 2004; Purro et al., 2012), and SAMP8 AD model (Bayod et al., 2015). DKK-1 gene is also a transcriptional target of tumor-suppressing protein p53 in mammalian cells (Wang et al., 2000). Thus, the induction of DKK-1 may represent a component of the sequential events for leading to neuronal death, and the activation of Wnt signaling could be an appropriate target for the treatments of AD.

Regular physical activity is beneficial for brain health and cognitive capacity. Whether Wnt signaling is also abnormal in natural aging rats and exercise has beneficial effect on Wnt signaling and corresponding targets is still unknown. Therefore, the aim of this study is to ascertain the effects of ladder climbing (one kind of resistance exercise models), treadmill running, and alternating exercise with ladder climbing and treadmill running, on brain aging in naturally aged rats by evaluating AD-like pathological proteins, synaptic plasticity, and aging-related biomarkers. Wnt/β-catenin signaling, a critical molecule related to cognition and neuronal survival was also evaluated to uncover the possible mechanisms for optimal exercise modes to mitigate brain aging.

## Materials and Methods

### Reagents

Primary antibodies including PHF10, p-Tau, Tau, SYN, PSD-93, PSD-95, p-AKT^ser473^, AKT, GSK-3β, p-GSK-3β^ser9^, β-catenin^Ser33, Ser37, Thr41^, active β-catenin, β-catenin, Axin1, Bcl-2, Bax, Ac-p53, p53, Sirt1, and GAPDH, as well as secondary antibodies were purchased from Cell Signaling Technology (Danvers, MA, USA).

### Animal Grouping and Treatments

Totally 50 male Wistar rats (age: 6 months old; body weight: 160 ± 20 g; No.: 42000600020737) were purchased from the Experimental Animal Center of Hubei Provincial Disease Control Center (Wuhan, China). The experimental protocols were approved by Institutional Animal Care and Use Committee at Wuhan Sports University. The natural aging rat model was established through feeding the rats from 6 months old to 21 months old. The aging model rats at the age of 21 months old were randomly divided into four groups including natural aging model group (OC), natural aging combined with resistance exercise group (OR), natural aging combined with treadmill training group (OT), and natural aging combined with alternating exercise with resistance exercise and treadmill running group (OM), with 10 rats in each group. The rats with the age of 6 months old were used as the young control group (YC). All animals were housed at the environment with room temperature of 22 ± 2°C and a dark-light cycle (12 h: 12 h), and provided with the accessibility to foods and water ad libitum. After 1 week adaptation, the rats from OR, OT and OM groups were subjected to corresponding exercise intervention once a day at 8:00 pm for 12 consecutive weeks.

### Experimental Protocols

#### Ladder-Climbing Training Protocol

Ladder-climbing intervention was implemented using the existing ladder of the laboratory, as described previously (Tang et al., 2014). The training time was selected in the dark cycle of the rats (18:00–20:00) with 3 days/week (once every other day). Briefly, the rats with heavy burden attached to their tails climbed a 1-m ladder with 2-cm grid and inclining at 85° defined as the resistance exercise. The rats in the first week were subjected to heavy burden for 10% of their body weights and gradually increased according to 10% of body weights every week during 12-week training period. At the last week, the attached heavy burden was 80% of their body weights. When the rats reached the top of the ladder, they were allowed to rest for 1 min in a dark area. The ladder-climbing intervention includes two sets of three ladder-climbing repetitions and 2 min rest interval during each set.

#### Treadmill Running Protocol

The rats were subjected to treadmill running without weight-bearing burden. The training time was selected in the dark cycle of the rats (18:00–20:00) with 3 days/week (once every other day). In order to adapt to the apparatus and avoid subsequent exercise-induced stress, the rats were placed on the treadmill for 30 min without running on the first day. The rats from OT group started running at a speed of 4.2 m/min, and gradually increased by 1 m/min every 30 s up to a speed of 12 m/min (between the 3rd minute and the 12th minute). The rats from young control group were placed individually on another treadmill (0 m/s) for same session number and duration as the YC group. The duration of treadmill running was 60 min. Neither electrical shock nor physical prodding was used to force running in these training stage.

#### Alternating Exercise With Ladder-Climbing and Treadmill Running

The total exercise intervention time was the same as above protocols, but resistance exercise and treadmill running were rotated every day.

### Sample Harvesting of Hippocampal Tissues From the Rats

After subjected to corresponding exercise interventions, the rats were sacrificed by decapitation, and their brains were dissected on ice to harvest hippocampal tissues. The harvested hippocampal tissues were immediately frozen in liquid nitrogen and then transferred to −80°C freezer for further analysis.

### Behavioral Testing

After different exercise interventions for 12 weeks, Morris water maze (MWM) test was used to assess spatial learning and memory capacity of the rats from different groups. MWM is consisted of a circular pool (150 cm in diameter) filled with water. A platform was submerged 2 cm deep in water in one of four identical quadrants. The duration of MWM test was 5 days. The first day represented the positioning test. The rats were released into water at different starting points and subjected to four trials each day to find the platform. The rats failed to find the platform within 120 s were gently guided to the platform and allowed to stay on the platform for recording. At the same time, the escape latency was also recorded. On the 6th day, the platform was removed. Rats were allowed to swim freely for 120 s. The latency to crossing platform, the time spent in the target quadrant and the number of crossing target platform were recorded.

### Preparation and Histological Examination of Hippocampal Tissues

After MWM task, five rats from every group were decapitated under anesthesia. Brain tissues were immediately harvested, and hippocampal tissues were isolated and frozen in liquid nitrogen for future analysis. Moreover, five rats were anesthetized and perfused with 0.9% saline followed by 4% paraformaldehyde (pH 7.4). Brain was removed and immersed in 4% paraformaldehyde at 4°C overnight, and then subjected to sequential paraffin embedding and sectioning at a thickness of 4 μm. At the same time, the samples of hippocampal tissues were harvested at the volume of 1 mm^3^, fixed in phosphate buffer containing 2.5% glutaraldehyde for 2 h, and then rinsed with 1 mmol/L phosphoric acid solution and fixed in 1% osmium tetroxide for 2–3 h. The block was cut carefully into ultrathin sections at the thickness of approximately 70 nm. The sections were stained with 3% uranyl acetate and lead citrate and then examined under a transmission electron microscope (TEM, HT7700, Hitachi, Japan) at Research Center for Medicine and Structural Biology of Wuhan University.

### Western Blot Analysis

Hippocampus samples were homogenized in lysis buffer (20 mM Tris, 135 mM NaCl, 2 mM EDTA, 2 mM DTT, 25 mM β-glycerophosphate, 2 mM sodium pyrophosphate, 10% glycerol, 1% Triton X-100, 1 mM sodium orthovanadate, 10 mM NaF, 10 μg/ml aprotinin, 10 mg/ml leupeptin, and 1 mM PMSF) for 30 min on ice. The lysates were centrifuged at 15,000× *g* at 4°C for 10 min. The protein samples were boiled at 95°C water bath for 5 min. The soluble protein was subjected to SDS-PAGE, and then transferred to nitrocellulose membrane. The target protein was probed by corresponding primary and secondary antibodies. Finally, the target protein was visualized by enhanced chemiluminescence (ECL) reagent and imaged by ultra-sensitive fluorescence/chemiluminescence imaging system ChemiScope6300 (CLiNX Science Instruments, Shanghai, China).

### Statistical Analysis

All data were expressed as mean ± standard deviation (M ± SD). Statistical analysis for single comparison was conducted by Student’s *t*-test and the statistically significant difference was considered at *p* < 0.05.

## Results

### Exercise Attenuated Dysfunctional Cognition of Aged Rats

**Figure 1:**
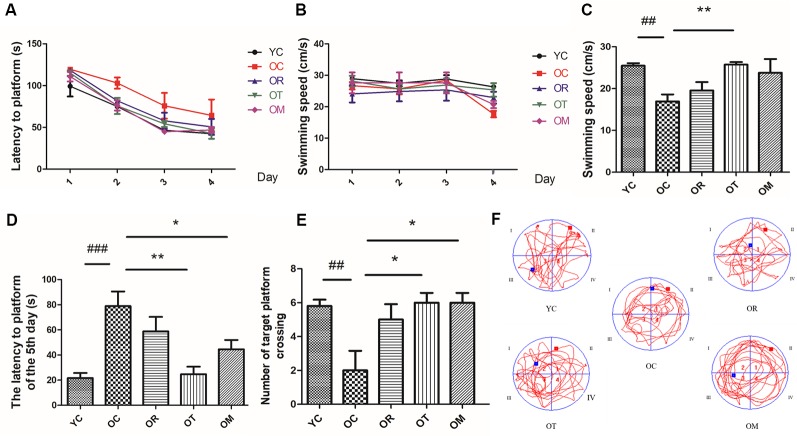
The learning and memory capacity in rats from different groups was assessed by Morris Water Maze (MWM; *n* = 10 per group). **(A)** Latency to platform of the rats during 1–4 days MWM training. **(B)** Swimming speed of the rats during 1–4 days MWM training. **(C)** Swimming speed of the rats on the 5th day. **(D)** Latency to platform of the rats on the 5th day. **(E)** Number of crossing target platform. **(F)** Swimming track. ^##^*p* < 0.01 and ^###^*p* < 0.001 relative to the YC group; *p < 0.05 and **p < 0.01 relative to the OC group.

Progressive decline in learning and memory capacity is an important clinical feature of AD so that we assessed potential neuro-protective effects of different exercise interventions on neurotoxicity using MWM test in naturally aged rats through evaluating the spatial learning and memory capacity. As shown in Figures 1A,D, the escape latency of the rats from OC group was significantly prolonged, while the rats from OR, OT and OM groups exhibited significantly shortened escape latency. In the MWM experiment, the mean swimming speed of the rats from different groups revealed no obvious difference during 1–4 days MWM training (Figure 1B), but on the 5th day, as shown in Figure 1C, the mean swimming speed of the rats from OC group was significantly decreased; in contrast, markedly increase in the OT group. As shown in Figures 1E,F, compared with YC group, the number of crossing platform in OC group revealed a significant decrease, indicating the decline of learning and memory capacity. Meanwhile, the rats from OT and OM groups markedly increased the number of crossing platform position when compared with the rats from OC group. These results indicated that the natural aging rats could result in decreased cognition and different exercise interventions could significantly improve their learning and memory capacity.

### Exercise Mitigated the Damage of Hippocampal Neurons in Aged Rats

**Figure 2:**
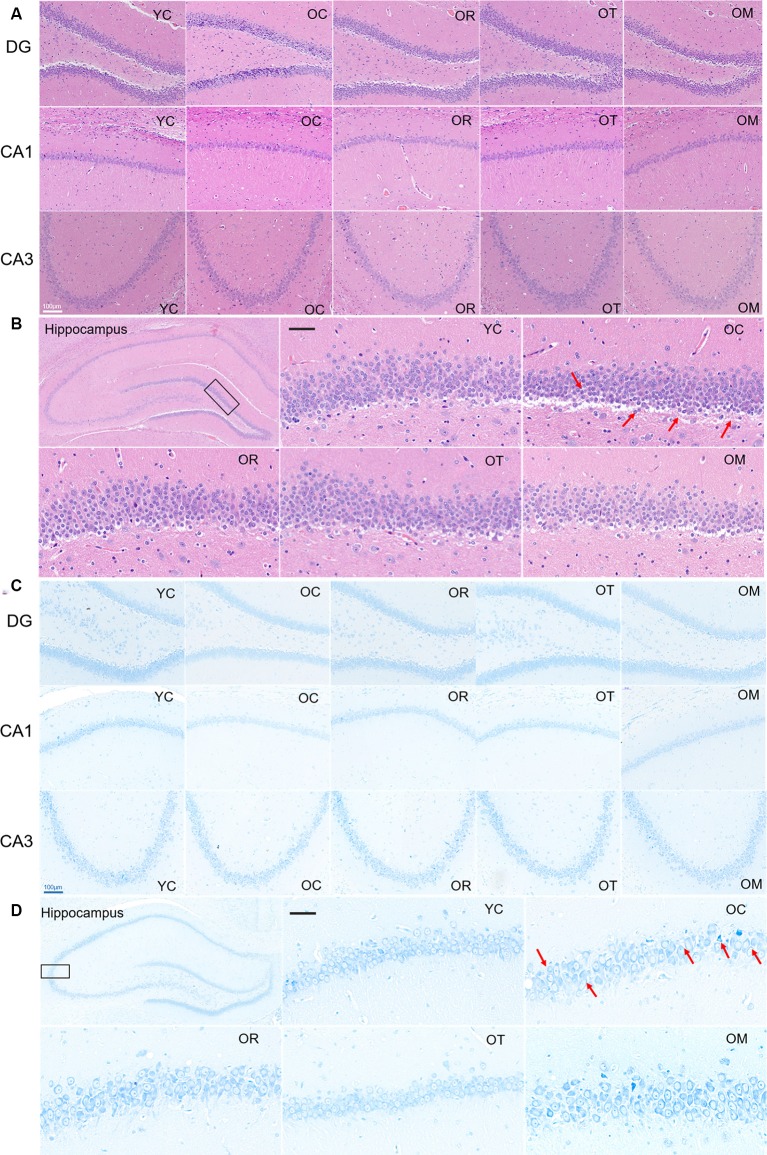
Representative photomicrographs demonstrating histopathological changes in hippocampal tissues with HE staining [(**A,B)**; scale bar, 50 μm] and Nissl staining [**C,D)**; scale bar, 100 μm]. Photographs from panels **(B,D)** were acquired under a light microscope (400×). All arrows in the images denoted the disordered neurons with nucleus condensation in the rats from OC group when compared with the normal hippocampal neurons in the rats from YC group and OR, OT and OM groups.

In order to evaluate the damage of hippocampal neurons in aged rats, histopathological changes were examined by HE and Nissl staining. Compared with the rats from YC group, the neurons in the hippocampal CA1 and CA3 regions of the rats from OC group were disorderly arranged, the number of neurons was reduced, and the number of apoptotic neurons was significantly increased. However, the rats subjected to OT and OM interventions exhibited a significant decrease in damaged neurons, suggesting that both kinds of exercise interventions can effectively attenuate the damage of hippocampal tissues in aged rats (Figure 2A). Moreover, compared with the rats from YC group, the neurons in hippocampal subfield of the rats from OC group were markedly decreased and damaged or lost in three different regions of hippocampal tissues (Figures 2B–D); in contrast, the hippocampal tissues in the aged rats from OR, OT and OM groups displayed the more regularly arranged, deeply stained and normal neurons when compared with OC group, indicating that exercise can ameliorate neurodegeneration of aged rats.

### Exercise Inhibited the Expression of DKK-1 and Senescence-Related Proteins in Hippocampal Tissues of Aged Rats

**Figure 3:**
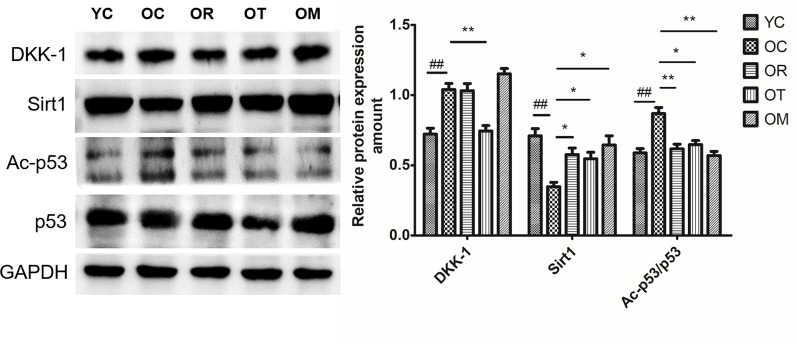
The effect of different types of exercise interventions on protein expression of DKK-1, Sirt1, Ac-p53 and p53 in hippocampal tissues of naturally aged rats. DKK-1, Sirt1, Ac-p53 and p53 protein expression was evaluated by Western blot. The data were expressed as mean ± standard deviation (M ± SD) from independent experiments performed in triplicate. Equal protein loading was confirmed by GAPDH. ^##^*p* < 0.01 and ^###^*p* < 0.001 relative to the YC group; *p < 0.05 and **p < 0.01 relative to the OC group.

To investigate Wnt signal pathway in hippocampal tissues after 12-week exercise interventions, its antagonist DKK-1 was assessed by Western blot. As shown in Figure 3, the expression level of DKK-1 in hippocampal tissues of the aged rats was significantly increased when compared with the young control rats, suggesting increased DKK-1 with the extension of age. Due to the regulation by its substrate p53 and Sirt1 (Wang et al., 2000; Caricasole et al., 2004; Hussain et al., 2009), the decreased expression of Sirt1 and significantly increased expression of acetylated p53 (Ac-p53) and p53 in neurons of hippocampal tissues from OC rats with respect to YC rats were observed (Figure 3); however, 12-week treadmill running markedly decreased the expression of DKK-1 protein, and interestingly, three different exercise interventions inhibited aging-induced up-regulation of Ac-p53 and p53.

### Exercise Ameliorated Synaptotoxicity Triggered by Aging Process

**Figure 4:**
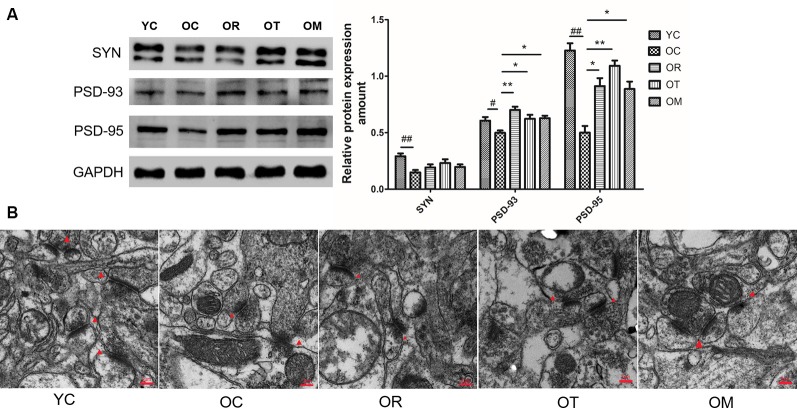
The effect of different exercise intervention on synaptotoxicity and synaptic morphology in hippocampal tissue. **(A)** SYN, PSD93 and PSD95 were subjected to Western blot analysis using corresponding antibodies. The data were expressed as mean ± standard deviation (M ± SD) from independent experiments performed in triplicate. Equal protein loading was confirmed by GAPDH. **(B)** Representative images of hippocampal tissues were examined by transmission electron microscope. The effect of exercise intervention on synapse (red triangle arrows) is indicated. ^#^*p* < 0.05, ^##^*p* < 0.01 relative to the YC group; *p < 0.05 and **p < 0.01 relative to the OC group.

Synaptophysin is a biomarker of cerebral plasticity. In order to evaluate the synaptic integrity in different exercise intervention groups, protein markers including synaptophysin (SYN), PSD-93 and PSD-95 (postsynaptic density protein) associated with specific synaptic plasticity were analyzed by Western blot. Our data showed that the expression levels of SYN, PSD-93 and PSD-95 were sharply reduced in hippocampal tissues of OC rats when compared with the rats from YC group, indicating that DKK-1 up-regulation significantly affected synapses (Figure 4A). However, three different types of exercise interventions could result in a significant increase in PSD-93 and PSD-95.

In addition, the number of synapses was reduced and synaptic gaps revealed the remarkable diminish in the rats from OC group when compared with the rats from YC group, as observed by TEM. Moreover, the postsynaptic membrane was thickened in the rats from OC group (Figure 4B). However, exercise interventions increased synapse number and improved hippocampal network structure of OC rats.

### Exercise Attenuated Aging-Induced Reduction of p-CREB and BDNF

**Figure 5:**
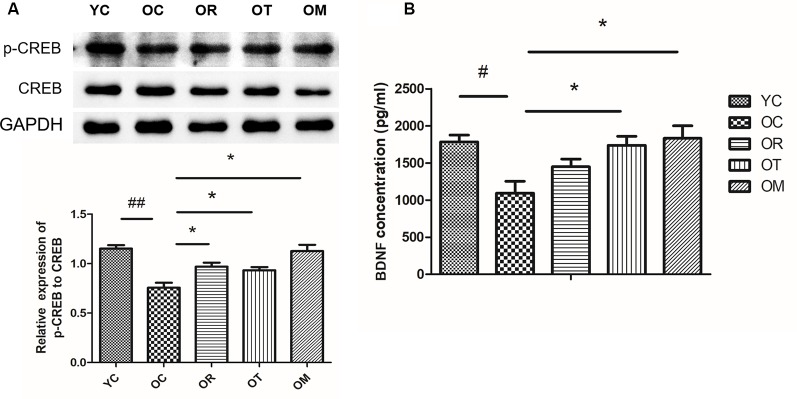
Exercise reversed the decrease of p-CREB/BDNF protein expression resulted from aging process. **(A)** p-CREB and CREB were evaluated by Western blot. **(B)** BDNF was determined by ELISA kit. Equal loading protein was confirmed by GAPDH. ^#^*p* < 0.05, ^##^*p* < 0.01 relative to the YC group; *p < 0.05 relative to the OC group.

In order to confirm whether the deletion or decline of neurotrophic factors is involved in memory impairment in aged rats, we detected brain-derived neurotrophic factor (BDNF) and cAMP response element-binding protein (CREB) as the crucial functional proteins for memory formation and consolidation (Benito and Barco, 2010). As shown in Figure 5A, p-CREB was different in hippocampal tissues from four groups. Compared with the young control rats, p-CREB was significantly decreased in OC rats, but increased upon three exercise interventions. In addition, the release of BDNF in hippocampal tissues was further assessed by ELISA. Both treadmill running and alternating exercise with resistance exercise could block aging-induced decrease in BDNF level when compared to sedentary aging controls (Figure 5B).

### Exercise Restored Down-Regulated Wnt Signal Pathway in Hippocampal Tissues of Aged Rats

**Figure 6:**
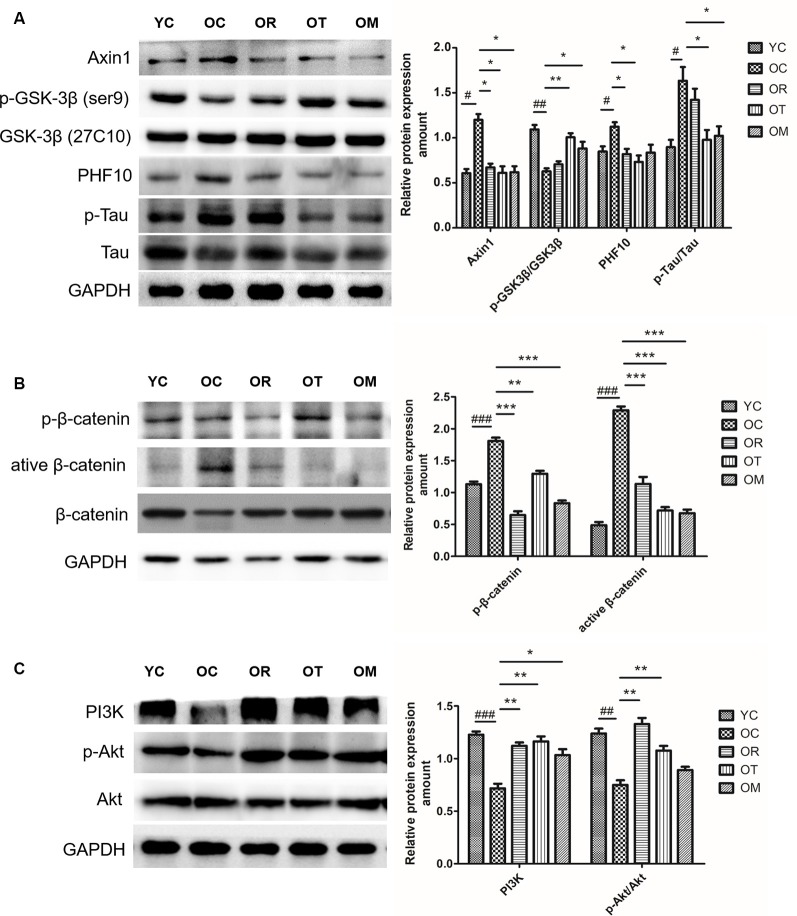
Protein expression levels of p-GSK-3β^Ser9^/GSK-3β, PHF-10, p-β-catenin, active β-catenin, β-catenin, PI3K, p-AKT and AKT in hippocampal tissues of the rats from different groups. **(A)** Western blot images and quantification of Axin 1, p-GSK-3β(ser9)GSK-3β, PHF10, p-Tau, Tau in hippocampus. **(B)** Western blot images and quantification of p-β-catenin, active β-catenin and β-catenin in hippocampus. **(C)** Western blot images and quantification of p-PI3K, p-AKT and AKT in hippocampus. Equal loading protein was confirmed by GAPDH. ^##^*p* < 0.01 and ^###^*p* < 0.001 relative to the YC group; *p < 0.05, **p < 0.01 and ***p < 0.001 relative to the OC group.

GSK-3 is a key component of Wnt signaling pathway (Doble et al., 2007). Previous study has revealed that GSK-3β accounts for several features of AD such as memory impairment, synaptic failure, Tau hyperphosphorylation and neuronal death (Hooper et al., 2008). Axin1 is a scaffold protein that promotes GSK-3β-mediated phosphorylation. In the present study, higher Axin1 level was measured in the rats from OC group. As us expected, three exercise interventions decreased the expression of Axin1. Since GSK-3β is negatively regulated (inactivated) by phosphorylation at site of Ser9, similar with DKK-1, the lower expression level of p-GSK-3β^Ser9^ in the rats from OC group was detected. However, three exercise interventions up-regulated the expression of p-GSK-3β^Ser9^ (Figure 6A). Moreover, GSK-3 activation in aged rats from OC group was accompanied by an increase in Tau phosphorylation when compared with the rats from YC group. In contrast, exercise interventions except OM ameliorated p-Tau level.

The impairment of Wnt ligands is correlated with the presence of DKK-1, and GSK-3β is activated and able to induce p-β-catenin^Ser33, Ser37, Thr41^, thus resulting in ubiquitylation and proteasomal degradation. In order to determine whether exercise can affect β-catenin phosphorylation by regulating GSK-3β activation, higher expression level of p-β-catenin^Ser33, Ser37, Thr41^ in OC rats was examined by Western blot when compared with YC rats (Figure 6B), suggesting that p-β-catenin^Ser33, Ser37, Thr41^ is closely correlated with aging process; on the other hand, exercise interventions significantly decreased p-β-catenin^Ser33, Ser37, Thr41^ level. Furthermore, the lower active catenin (unphosphorylated β-catenin^Ser37, Thr41^, ABC) level in OC rats was observed as compared with the rats from YC group, but total β-catenin protein level did not reveal an obvious change. Taken together, our findings suggest that DKK-1 can induce a down-regulation of Wnt signaling in hippocampal tissues of aged rats.

PI3K/Akt/GSK-3β signal pathway is altered in AD brain (Jimenez et al., 2011). Therefore, we further evaluated the effect of exercise on PI3K/Akt signal pathway. The significantly decreased PI3K and phosphorylated Akt levels in OC rats were observed when compared with YC rats, and total Akt protein level remained unchanged among different exercise intervention groups with various age. In contrast, resistance exercise and treadmill running could significantly increase Akt phosphorylation (Figure 6C).

### Exercise Attenuated Neuronal Apoptosis Associated With Inhibited Wnt Signal Pathway in Hippocampal Tissues of Aged Rats

**Figure 7:**
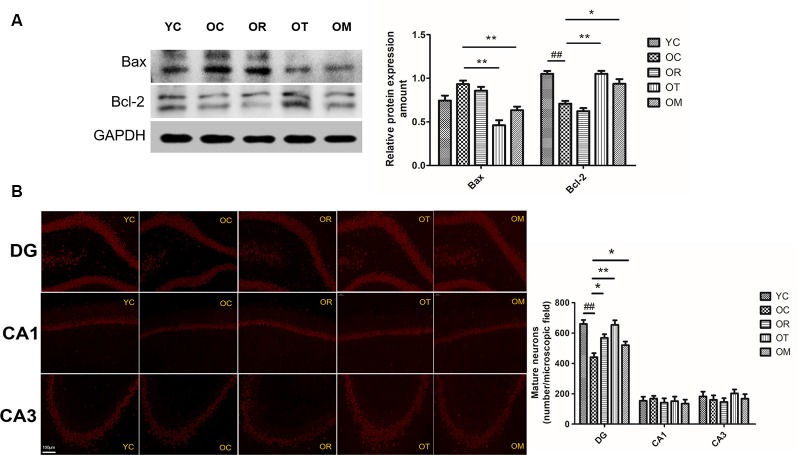
Protein expression levels of Bax and Bcl-2 in hippocampal tissues of the rats from different groups. **(A)** Images showed representative Western blots and bar graphs obtained from semi-quantitative image analysis. All data were presented as mean ± standard deviation (M ± SD) from independent experiments performed in triplicate. **(B)** Representative images of dentate gyrus (DG), CA3, and CA1 regions stained with NeuN from brains of the rats from different groups (200× magnification). ^##^*p* < 0.01 relative to the YC group; *p < 0.05 and **p < 0.01 relative to the OC group.

The regulation of Bax and Bcl-2 by Wnt signal pathway has been reported. So, the impairment or inhibition of Wnt signal pathway by DKK-1 is correlated with higher Bax level and lower Bcl-2 level. In addition, the activation of Wnt signal pathway can up-regulate Bcl-2 (Fuentealba et al., 2004). Herein, higher Bax level and lower Bcl-2 level in hippocampal tissues of OC rats were observed when compared with the rats from YC group (Figure 7A). On the contrary, both treadmill running and alternating exercise with ladder climbing and treadmill running markedly reduced Bax level and promoted the expression of Bcl-2.

As shown in Figure 7B, reduced NeuN immunostaining in different hippocampal areas (CA1, CA3 and dentate gyrus (DG) regions) from OC rats was also observed when compared with the rats from YC group, indicating the loss of mature neurons. In contrast, three exercise interventions ameliorated the loss of mature neurons resulted from aging.

## Discussion

In the current study, whether Wnt signaling can be down-regulated and its possible relationship with neuronal apoptosis in hippocampal tissues of naturally aged rats were investigated. Meanwhile, whether resistance exercise, treadmill running and alternating exercise with ladder climbing and treadmill running can rescue or partially rescue cognitive dysfunction by modulating Wnt signaling was also explored. Similar with previous studies (Bayod et al., 2015), a significant down-regulation of Wnt signaling in OC rats is observed, together with increased AD-like pathological protein level, synaptotoxicity and neuronal loss. As expected, three kinds of exercise interventions can promote the rescuing of cognitive dysfunction partly by restoring Wnt signaling pathway.

DKK-1 is one of the most extensively studied Wnt antagonists. One study has reported that long-term moderate treadmill running can activate Wnt signaling of hippocampal tissues in normal adult male rats (Bayod et al., 2014). Higher expression level of DKK-1 is detected in different AD models and temporal dementia as well as AD patients (Caricasole et al., 2004; Rosi et al., 2010). DKK1 level in hippocampal tissues of mice reveals an increase with age extension, accompanied by the impairment of spatial memory; however, the knockdown of DKK1 in hippocampal tissues improves the spatial memory of mice, thereby reversing the aging-induced memory impairment (Seib et al., 2013). Therefore, DKK1 plays a key role in influencing cognitive function through Wnt signaling. In addition, Sirt1 plays critical roles in a series of brain functions; as confirmed by one of previous studies with the significant down-regulation of Sirt1 in oxidative stress-induced AD rats and obvious up-regulation of Sirt1 upon 8-week swimming intervention, thereby improving cognitive function (Kou et al., 2017). Due to the cross-talk among DKK-1, Sirt1 and p53, we have also determined the relationships among DKK-1, Sirt1 and p53 in hippocampal tissues of aged rats. Accordingly, a close correlation between higher expression of DKK-1 and lower expression of Sirt1, as well as increased Ac-p53/p53 ratio in hippocampal tissues of OC rats is observed. Herein, treadmill training can decrease DKK-1; in turn, enhance Sirt1 and inhibit Ac-p53 and p53, indicating that the up-regulation of Sirt1 is correlated with a reduction of Ac-p53. However, there are some inconsistent results regarding *Sirt1* gene expression in brain tissues from different AD models. No change of Sirt1 protein expression in the parietal cortex of 12–20-month-old 3xTg-AD mice has been reported (Julien et al., 2009), and some studies do not reveal the difference in Sirt1 level in AD patients when compared with the healthy elderly (Furuya et al., 2012). To our knowledge, these variances in Sirt1 may be linked with differential changes in the temporal expression pattern of Sirt1 protein or mRNA in 3xTg-AD mice.

The loss of synapses occurs prior to neural death. Synaptic loss and death of specific neurons in AD are provoked by a cascade of multiple deleterious molecular and cellular events. Recent studies have found that Wnt/β-catenin signaling is not only required for synapse formation, but also can regulate neurotransmission through acting both pre- and post-synaptically (McLeod and Salinas, 2018). One feature in aged rats from OC group in the present study is the decline of SYN, PSD-93 and PSD-95 triggered by aging, which plays a prominent role in the dysfunction of cognitive capacity. A growing body of studies has shown that exercise as an effective stimulus can cause plastic change in brain from structure to function, and from cells to molecules, thereby suppressing brain aging or achieving healthy aging of human body, with less cognitive impairment and less cognitive dysfunction. Our results clearly indicate that three kinds of exercise interventions can rescue the aging-induced reduction of PSD-93 and PSD-95, but not SYN. Brain science researches believe that the brain has plasticity throughout life, and exercise is an important strategy to stimulate its plasticity. It is generally accepted that different kinds of exercise models may affect different activities of certain parts of the body, and map specific areas of the brain, thus activating specific areas of the brain. We speculate that the non-response of exercise to SYN may be related to different exercise modes. During the studies of exercise and brain function, more studies are mainly focused on exercise and executive function, cognition function and memory function. As shown in Figures 1C,D, on the 5th day, the escape latency of the rats from OC group is significantly longer than that in the YC group. The mean swimming speed is also declined in the rats from OC group. In contrast, treadmill running and alternating exercise with ladder climbing and treadmill running can significantly shorten the escape latency when compared with OC group. Interestingly, only treadmill running can improve swimming speed. Similar with these results, the rats subjected to treadmill running and alternating exercise with ladder climbing and treadmill running also reveal the remarkably increased number of crossing platform when compared with the rats from OC group. From above results, we can conclude that the declined spatial learning and memory capacity of aged rats can effectively rescued by treadmill running intervention as an aerobic exercise. Meanwhile, these findings further demonstrate that the induction of DKK1 in hippocampal tissue caused by aging triggers synapse loss, synaptic dysfunction and memory impairment, all of which can be partially restored by up-regulating Wnt/β-catenin signaling upon different kinds of exercise interventions. The balance between Bcl-2 and Bax determines the survival or death of cells. In addition, previous studies have also demonstrated that DKK-1 induction has been found to precede neuronal death in AD models (Seib et al., 2013). Considering the transcription of DKK1 with the function of triggering apoptosis, we next have measured the expression of Bax and Bcl-2 proteins in OC rats. Similar with SAMP8 mice at the age of 9–12 months old, an increase in Bax expression together with lower Bcl-2 expression is observed. Moreover, the reduced NeuN immunostaining of the DG area in hippocampal tissue of OC rats also demonstrates an increase in mature neuronal loss. Axin1 is the key target of Wnt signaling. The inducible expression of Axin1 in hippocampal tissues of OC rats further supports the concept that the suppression of Wnt signaling can trigger the degeneration of hippocampal neurons. Interestingly, on the one hand, treadmill running and alternating exercise with ladder climbing and treadmill running can significantly increase Bcl-2 protein level, and decrease Bax protein level in OC rats. On the other hand, three kinds of exercise interventions can reverse neuron loss caused by aging at varying degrees. The most obvious efficiency for rescuing neuron loss by treadmill exercise is observed, as shown in Figure 7.

GSK3β is an important kinase associated with hyperphosphorylation of Tau (p-Tau) at corresponding phosphorylation sites. The classic “GSK-3 hypothesis in AD” proposes that the over-activation of GSK-3β is closely associated with following pathological features including memory impairment, increased Aβ production, Tau phosphorylation and neuronal death (Balaraman et al., 2006). GSK-3β overexpression in a conditional transgenic model could produce Tau hyperphosphorylation and neuronal death (Lucas et al., 2001; Engel et al., 2006). On the contrary, the inhibition of GSK-3β activity by lithium can reduce Tau-dependent pathology, and improve the performance in memory capacity in AD-Tg models (Su et al., 2004). DKK1 is able to inhibit Wnt/β-catenin signaling and induce the hyperphosphorylation of Tau (Scali et al., 2006). As mentioned previously, the inhibition of Wnt signaling by DKK-1 is associated with the increased GSK-3 activity. Therefore, in the current study, we have determined p-GSK-3β^Ser9^ in animals for different intervention groups. Consistent with DKK-1 results, an increase in p-Tau and a reduction of p-GSK-3β^Ser9^ (inactive form) are detected in OC rats. However, treadmill running not only can attenuate GSK-3β activity, but also can decrease p-Tau and PHF10 protein level. GSK-3β activity and p-Tau protein level exhibit a decrease in OM group although ladder climbing only reveals a decrease in PHF10. Consequently, treadmill training is one of the best intervention methods at this situation. Our data support the hypothesis that the activation of Wnt/β-catenin signaling induced by exercise causes the decline of GSK-3β activity and subsequent inhibition of p-tau is a promising strategy in the treatment of brain aging or AD. Currently, some GSK-3β inhibitors have been shown to inhibit p-Tau in different cell and animal models with AD (Llorens-Martin et al., 2014; Maqbool et al., 2016). However, the application of GSK-3β inhibitors in AD patients during clinical studies is disappointed due to the wide range of GSK-3β substrates and physiological actions. Another study has shown that DKK1 inhibitors can treat AD through inhibiting p-Tau protein caused by prostaglandin J2 (Mpousis et al., 2016). Recent studies have shown that curcumin can potentially activate Wnt/β-catenin signaling by increasing the expression of Wnt proteins and suppressing the expression of DKK1, but its practical application is still limited due to poor bioavailability in brain tissues (Farkhondeh et al., 2019; Sanei and Saberi-Demneh, 2019). Considering no specific inhibitors or activators in clinical practical application, our results raise the intriguing possibility that exercise intervention may avoid these harmful adverse effects from typical treatments.

As a target of GSK-3β, the phosphorylation of β-catenin can be mediated by GSK-3β, thereby resulting in ubiquitylation and proteasomal degradation. Next, we have sequentially explored whether p-β-catenin^Ser33, Ser37, Thr41^ can be affected in natural aging rat model. An increase of p-β-catenin^Ser33, Ser37, Thr41^ in OC rats is observed, thereby leading to its degradation by proteasome. Moreover, non-phosphorylated β-catenin reveals a weaker presence in hippocampal tissues of OC rats. Thus, higher phosphorylated β-catenin and a decrease in ABC, reinforces the notion that OC rats present the impairment of canonical Wnt/β-catenin signaling in hippocampal tissues. In contrast, three kinds of exercise interventions mitigate p-β-catenin^Ser33, Ser37, Thr41^ level and treadmill running is one of the best interventional strategies. Taken together, the reduction of Axin1 expression level and GSK-3β activation in rats from exercise intervention groups suggests an increase in nuclear translocation of β-catenin and the activation of Wnt signal pathway, thereby promoting neuronal survival. In this respect, one study has demonstrated that long-term treadmill training has no effect on p-β-catenin^Ser33, Ser37, Thr41^ and total β-catenin in 10-month-old rats. This inconsistency may be caused by different animal models. Age must also be considered for the onset of exercise interventions.

The transcription and expression of BDNF are mainly regulated by CREB. Previous studies have demonstrated that the increase in BDNF level in hippocampal tissues is specific to exercise (Kobilo et al., 2011; Vivar et al., 2013). We have found that the expression of BDNF, and p-CREB in hippocampal tissues of OC rats is significantly reduced, suggesting that the differential expression of BDNF and p-CREB is involved in the process of brain aging. Herein, BDNF level in the rats from OT and OM groups reveal a significant increase, while p-CREB protein level in rats from OR and OT groups is also promoted. Trophic factors improve neuronal survival largely through PI3K/Akt signaling pathway. After p-AKT activation, it can inhibit GSK-3β activation. Our current results demonstrate that abnormal expression of DKK1 not only alters the expression of the proteins and genes associated with canonical Wnt/β-catenin signaling pathway, but also changes other signaling pathways. We have found a decreased expression of PI3K and p-AKT protein in rats from OC group. Interestingly, compared with YC group, three kinds of exercise interventions, especially treadmill running, remarkably activate PI3K signal. Both treadmill running and ladder-climbing training also markedly up-regulate the expression of p-AKT, indicating the presence of increased neurotrophin in rats from OR, OT and OM groups.

In summary, the dysfunction of cognitive capacity, the decreased post-synaptic protein expression, the occurrence of AD-like pathological proteins, and the increased neuron apoptosis in rats are emerged with the extension of age. All of these changes are closely correlated with impaired Wnt signaling in hippocampal tissues of aged rats. However, three kinds of exercise interventions suppress negative effects on brain aging as the extension of age. Especially, treadmill training is better than other two kinds of exercise interventions. Therefore, moderate exercise is capable of activating Wnt signal pathway in hippocampal tissues of naturally aged rats. In addition, regulating PI3K/Akt signal pathway and increasing BDNF release may also participate in the process of mitigating brain aging.

## Data Availability Statement

The datasets generated for this study are available on request to the corresponding author.

## Ethics Statement

The animal study was reviewed and approved by Institutional Animal Care and Use Committee at Wuhan Sports University.

## Author Contributions

XK and NC conceived and designed the project. DC, YZ, MZ, JC, and ZZ performed the experiments. DC, YZ, MZ, JC, XK, and NC analyzed the data. DC, YZ, MZ, JC, and XK interpreted experimental results. DC, YZ, and XK prepared figures. DC, YZ, XK, and NC drafted the manuscript. XK and NC edited and revised the manuscript. DC, YZ, MZ, JC, ZZ, XK, and NC approved the final version of the manuscript.

## Conflict of Interest

The authors declare that the research was conducted in the absence of any commercial or financial relationships that could be construed as a potential conflict of interest.
